# Supplementary material for: Automated and optimally FRET-assisted structural modeling
Source: Nat Commun. 2020 Oct 26;11:5394. doi: 10.1038/s41467-020-19023-1 (PMC7589535; doi:10.1038/s41467-020-19023-1)
Supplement: Supplementary file 1 — Supplementary Information [file 41467_2020_19023_MOESM1_ESM.pdf]

Supplementary Information:
Automated and optimally FRET-assisted structural modeling

Mykola Dimura[1, 2], Thomas O. Peulen[1], Hugo Sanabria[1,3], Dmitro Rodnin[1], Katherina Hemmen[1], Christian A. Hanke[1], Claus A.M. Seidel[1, *], Holger Gohlke[2, 4, *]

[1] Chair for Molecular Physical Chemistry, Heinrich Heine University Düsseldorf, 40225 Düsseldorf, Germany;
[2] Institute for Pharmaceutical and Medicinal Chemistry, Heinrich Heine University Düsseldorf, 40225 Düsseldorf, Germany;
[3] Department of Physics and Astronomy, Clemson University, Clemson, South Carolina, U.S.A.
[4] John von Neumann Institute for Computing (NIC), Jülich Supercomputing Centre (JSC), and Institute of Biological Information Processing (IBI-7: Structural Biochemistry), Forschungszentrum Jülich GmbH, 52425 Jülich, Germany

**Supplementary Information**	**Page**
**Supplementary Figure 1**<br>Optimal FRET pair selection.	2
**Supplementary Figure 2**<br>Dependency of the reduced chi-squared value on the number of degrees of freedom	3
**Supplementary Figure 3**<br>Selection of conformers by FRET.	4
**Supplementary Figure 4**<br>FRET-guided NMSim simulations workflow.	5
**Supplementary Figure 5**<br>FRET-guided MD simulations workflow.	6
**Supplementary Figure 6**<br>FRET assisted modeling for two states of T4 Lysozyme.	7
**Supplementary Figure 7**<br>Correlation between the accuracy (RMSD of Cα atoms) and agreement with FRET (χ^2_n).	8
**Supplementary Figure**<br>Expected precision and FRET pair networks of benchmarked proteins.	10
**Supplementary Figure 9**<br>Calculation of expected precision.	11
**Supplementary Figure 10**<br>Optimal FRET pair selection algorithms.	12
**Supplementary Figure 11**<br>Measured FRET distances against predicted FRET distances for the best model	13
**Supplementary Table 1**<br>Selected FRET pairs and corresponding donor-acceptor averaged distances and errors for the target	14
**Supplementary Table 2**<br>List of primers used within this work.	15
**Supplementary Table 3**<br>Site-specific residual anisotropies in lysozyme	16
**Supplementary Note 1**<br>System selection and geometric modeling justification.	17
**Supplementary Note 2**<br>Dye models in the simulations	18
**Supplementary Note 3**<br>Local Distance Difference Test	19
**Supplementary Note 4**<br>Pseudocode for greedy FRET pair selection algorithm.	20
**Supplementary Note 5**<br>Pseudocode for greedy FRET pair elimination algorithm.	21
**Supplementary Note 6**<br>Pseudocode for mutual information-based FRET pair selection algorithm.	22
**Supplementary Note 7**<br>NMSim coarse-grained simulations	23

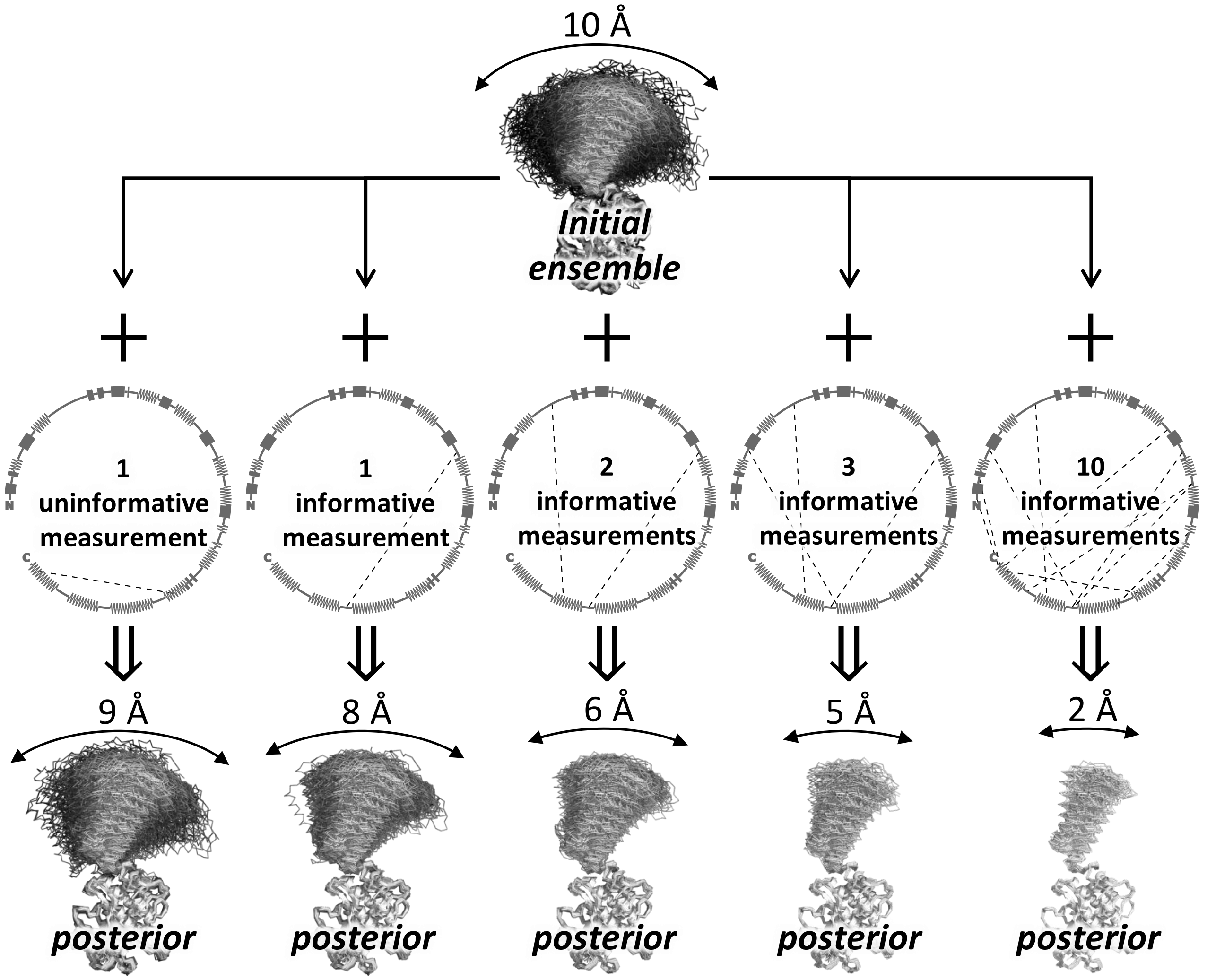

Supplementary Fig. 1 Optimal FRET pair selection.
At the top an exemplary initial conformational ensemble is depicted. The arrow over the ensemble reflects its structural diversity, the ⟨⟨RMSD⟩⟩ value is shown above. Circles in the middle row represent the secondary structure of the source protein conformation. Inside the circles, the set of FRET pairs is indicated by dashed lines. Given a pair set, the initial ensemble is narrowed (posterior, bottom row). More informative pairs lead to narrower posteriors. A larger pair set generally results in smaller ⟨⟨RMSD⟩⟩ as well. In the greedy forward feature selection algorithm, first, all possible donor-acceptor (DA) pairs are tested one by one, and the pair that yields the smallest posterior ⟨⟨RMSD⟩⟩ is selected. In the next iterations, remaining DA pairs are tested one by one, in order to determine, which additional pair in combination with pairs selected earlier will yield the smallest ⟨⟨RMSD⟩⟩. Thus, at each iteration, one optimal pair is added to the set, until the desired ⟨⟨RMSD⟩⟩ is reached or the number of required measurements is too high.

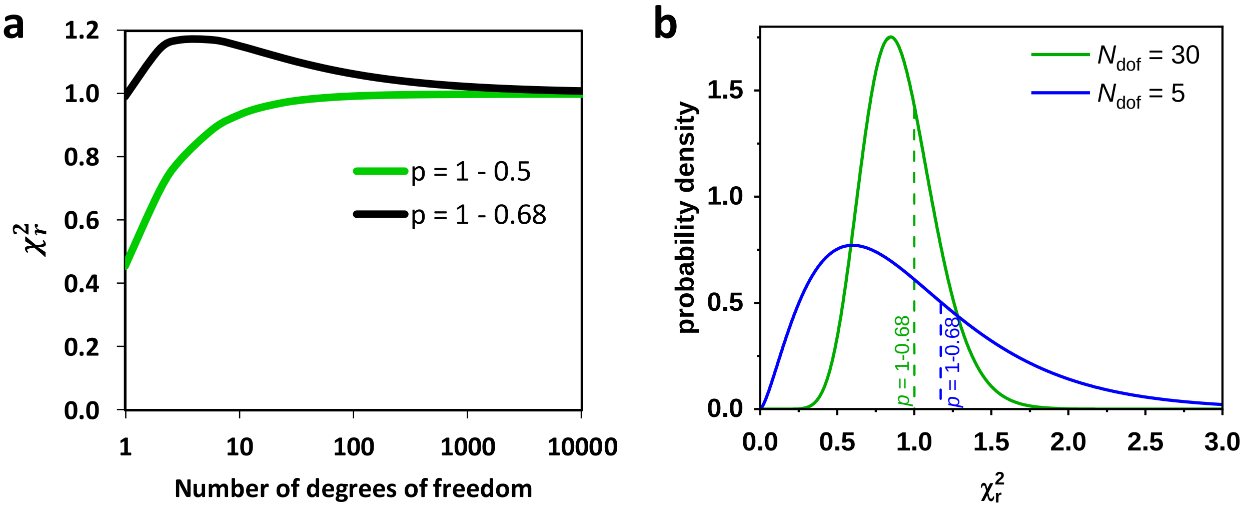

Supplementary Fig. 2 (a) Dependence of the reduced chi-squared value, χ_r^2, on the number of degrees of freedom for a constant value of confidence level. As illustrated, a constant confidence level corresponds to different χ_r^2 values, depending on the number of degrees of freedom in the test. **(b)** Example for a reduced chi-square distribution with 5 degrees of freedom (blue), and 30 degrees of freedom (green). Vertical dashed lines indicate models with confidence level of 68%. One can see, that for two models with the same statistical significance different χ_r^2 values are observed.

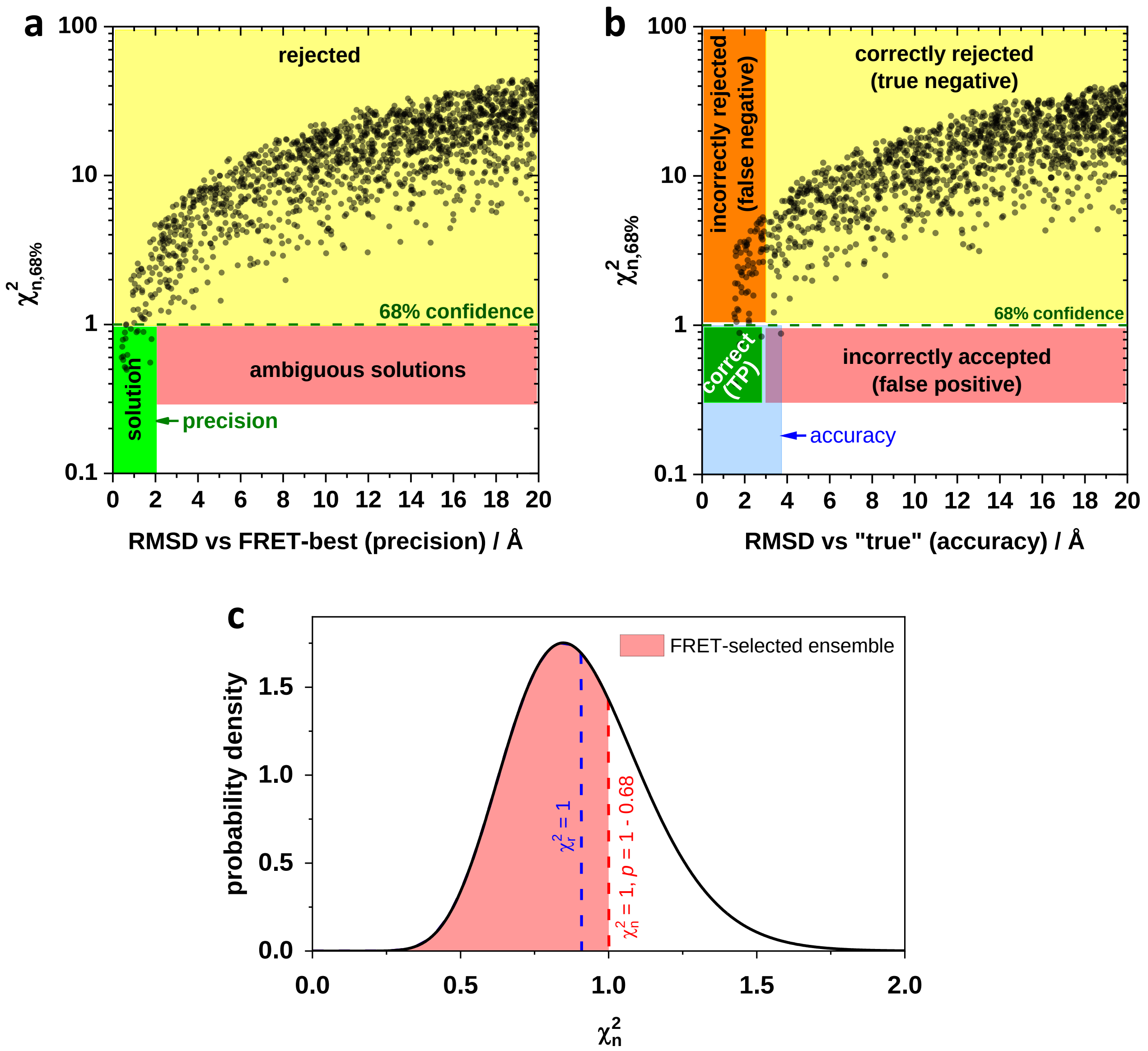

Supplementary Fig. 3 Selection of conformers by FRET.

On the y-axis, the normalized chi-squared reduced value, $\chi^2_{n,68\%}$, is shown. On the x-axis, the RMSD against the reference conformer is displayed. The horizontal line at $\chi^2_n = 1$ indicates the confidence level of 68%.

(**a**) The structure with the lowest χ^2_n is used as the reference for RMSD calculations. The RMSDs of the structures below the $\chi^2_n = 1$ threshold define (green box) the precision of the model. (**b**) The "true" (crystal structure) conformation is used as the reference for RMSD calculations. Here, RMSDs below the threshold define the accuracy of the model. The lower left corner of the plot shows correctly predicted structures (true positives, green box), conformers incorrectly selected by FRET (false positives, red box) would be on the lower right side, correctly discarded models (true negatives, yellow box) on the upper right side, and incorrectly discarded (false negatives, orange box) on the upper left side. **(c)** Chi-squared distribution probability density function. Conformers with $\chi^2_n < 1$ belong to FRET-selected ensemble. Red vertical dashed line indicates $\chi^2_n = 1$ value, blue vertical dashed line indicates $\chi^2_r = 1$.

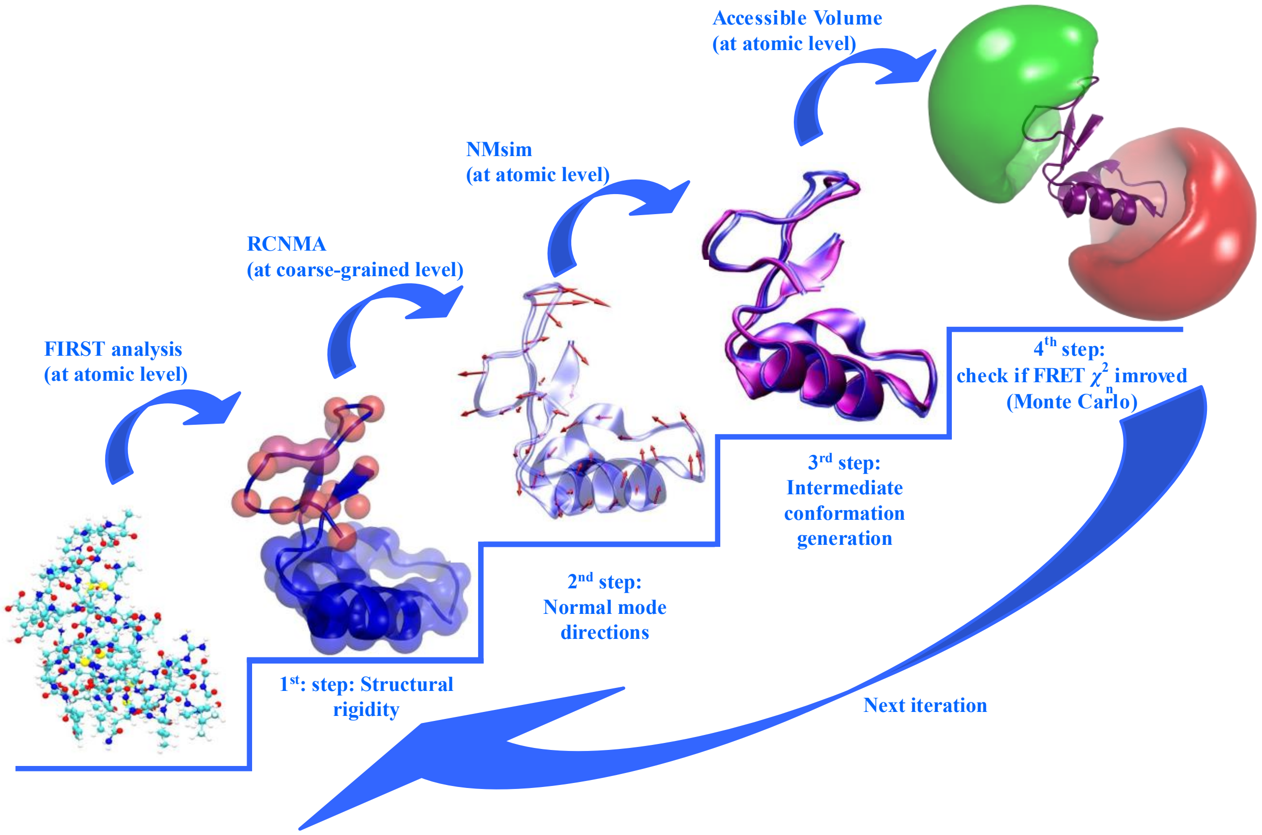

Supplementary Fig. 4 FRET-guided NMSim simulations workflow.
NMSim is a normal mode-based geometric simulation approach for multiscale modeling of protein conformational changes using three-step iterations: In the first step, the protein structure is coarse-grained by the software FIRST into rigid parts (colored blobs) connected by flexible links (single spheres). In the second step, low-frequency normal modes are computed by rigid cluster normal mode analysis (RCNMA). In the third step, a linear combination of the first normal modes is used to bias backbone motions along the low-frequency normal modes, while the side chain motions were biased towards favored rotamer states. The algorithm is here extended by a fourth step – a Markov Chain Monte Carlo step to prioritize conformations lying in most relevant regions according to the FRET χ_n^2 value. Depiction of steps 1 to 3 was adapted from Ahmed et al[1].

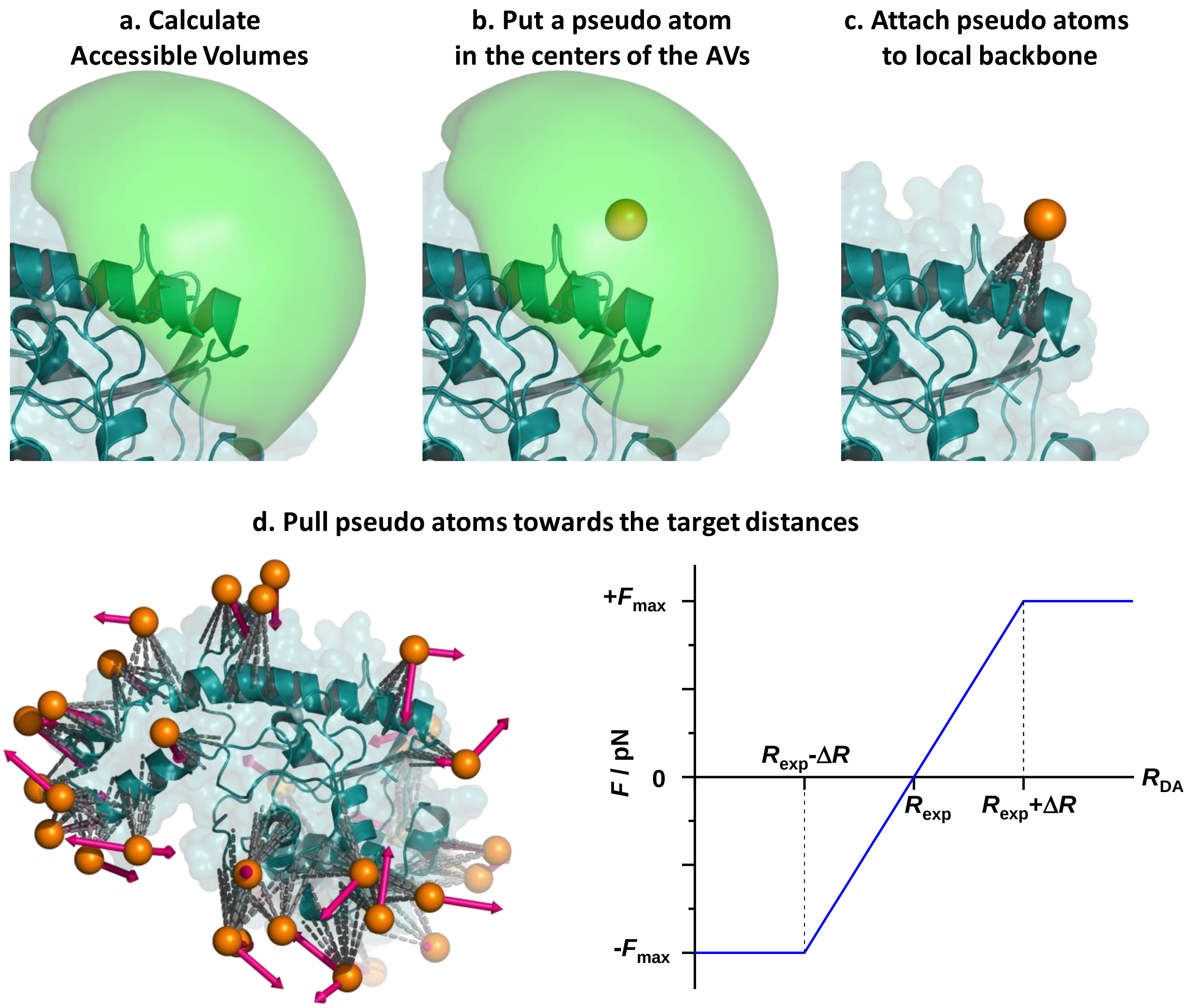

Supplementary Fig. 5 FRET-guided MD simulations workflow.
We introduce FRET restraints into MD simulations in a four-step approach (https://github.com/Fluorescence-Tools/FRETrest). **(a)** Accessible Volume (AV) calculations are performed for each labeling position. **(b)** Pseudo atoms are positioned at the mean position of every accessible volume. **(c)** Pseudo bonds (gray dashed lines) are created between the pseudo atom and nearby C_α and C_β atoms to keep pseudo atoms in their initial positions relative to the corresponding part of the protein backbone. **(d)** Restraints between pseudo atom pairs are applied to mimic measured FRET distances. To prevent unphysical unfolding of the protein, the FRET-restraint force is capped at an empirically determined value F_{max} = 50 pN, which is reached when the distance between pseudo atoms R_{DA} is more than one standard error (ΔR_{exp}) away from the optimum (R_{exp}).

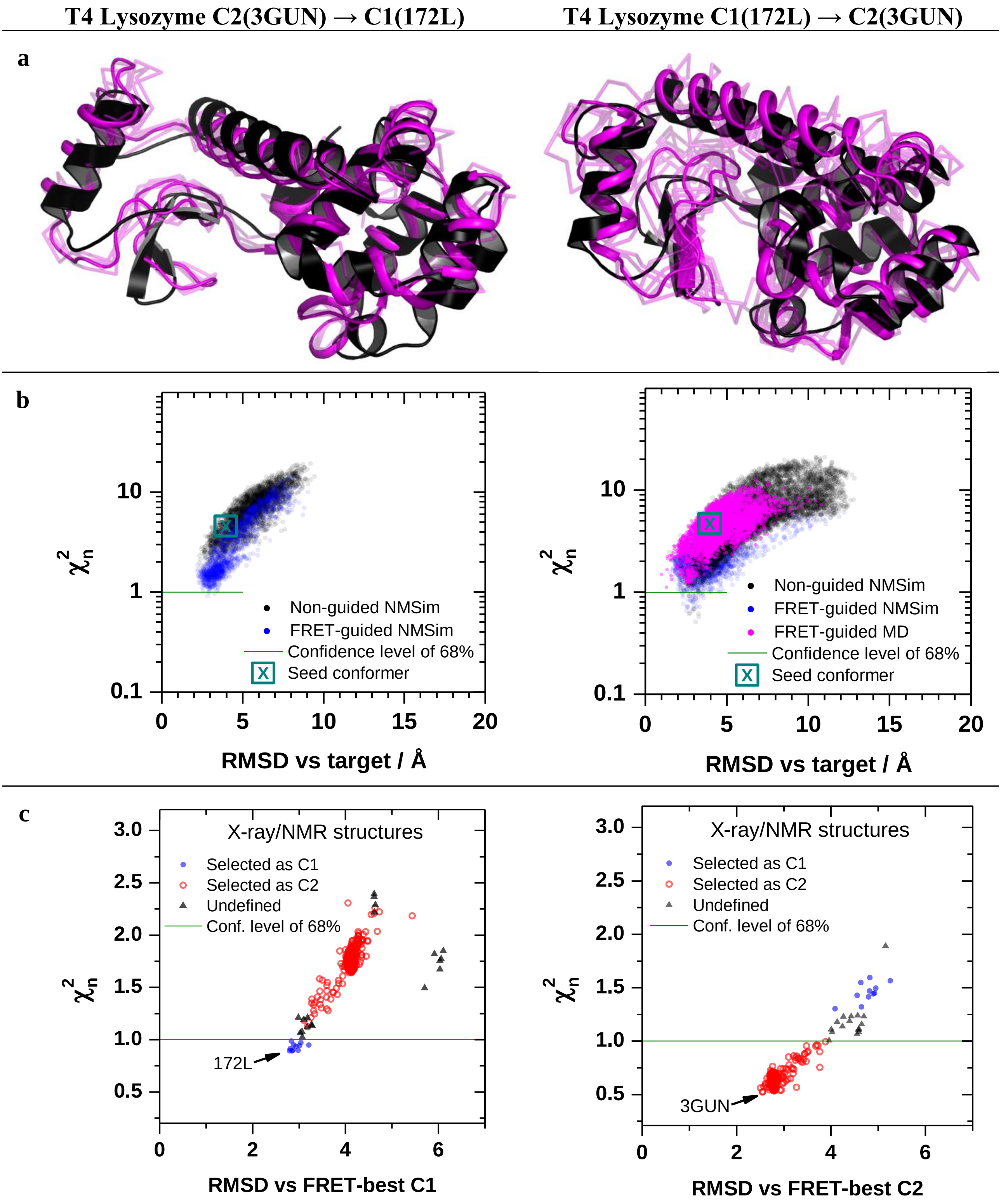

Supplementary Fig. 6 FRET-assisted modelling for two states of T4 Lysozyme. To the left, the C1 state obtained by FRET assisted modelling using a C2 crystal structure as a seed (C1→C2). To the right, the reverse situation (C2→C1) is shown: The C1 crystal structure serves as seed and the C2 conformation is determined by FRET assisted modelling. **(a)** FRET-selected ensemble with confidence level of 68%. **(b)** FRET χ_n^2 values and RMSDs against the crystal structure (target). Each point represents a conformation. Black points stand for unrestrained NMSim sampling starting from homology models. Blue points represent FRET-guided NMSim simulations. Magenta points represent FRET-restrained MD simulations. **(c)** FRET χ_n^2 values and RMSDs against the best FRET-based structure (lowest χ_n^2) for 571 X-ray and NMR structures from the PDB. Source data are provided as a Source Data file.

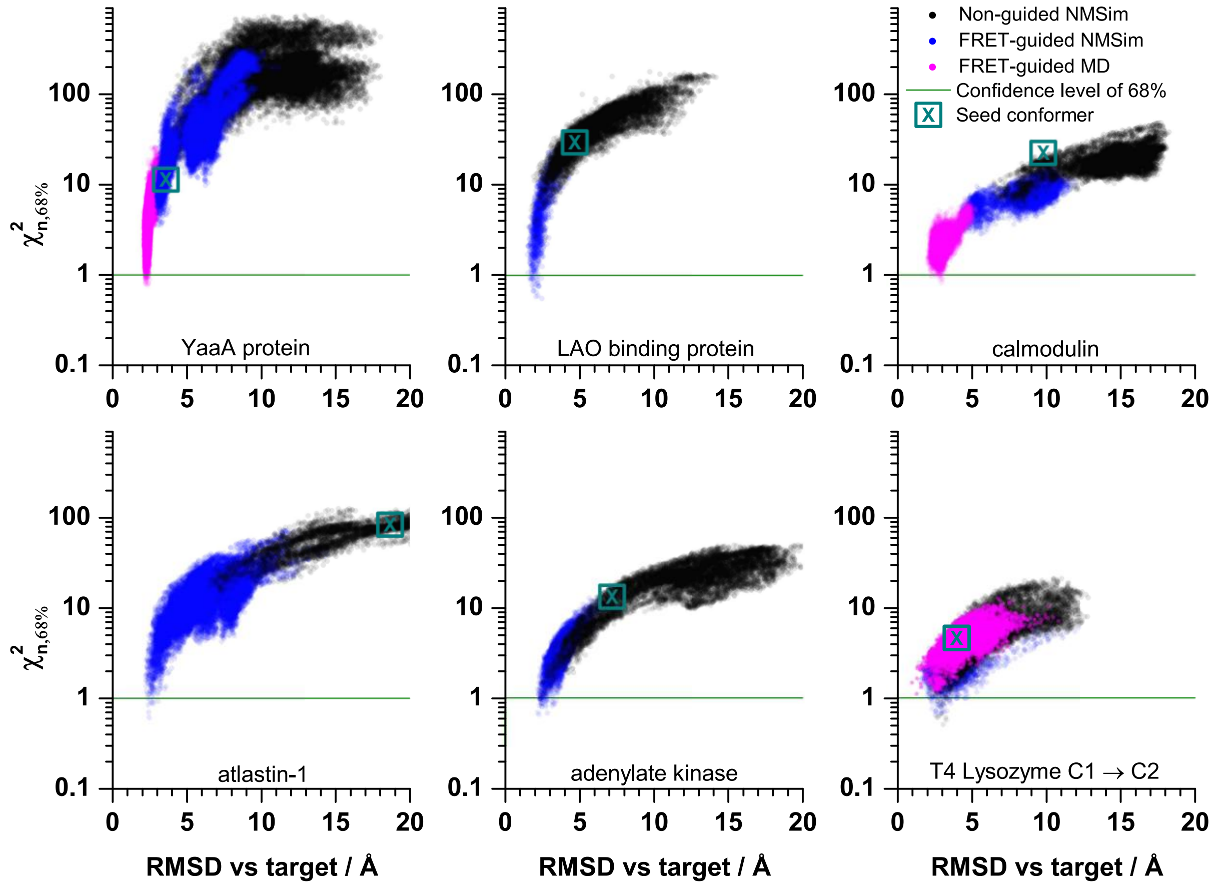

Supplementary Fig. 7 Correlation between the accuracy (RMSD of Cα atoms) and agreement with FRET (χ_n^2). Structures obtained from unrestrained NMSim simulations are shown as black dots, conformers from FRET-guided NMSim simulations are blue, and magenta represents the results of FRET-guided MD simulations. The confidence level of 68% is indicated by the green horizontal line. Seed conformers for each protein are indicated by cyan crosses. Source data are provided as a Source Data file.

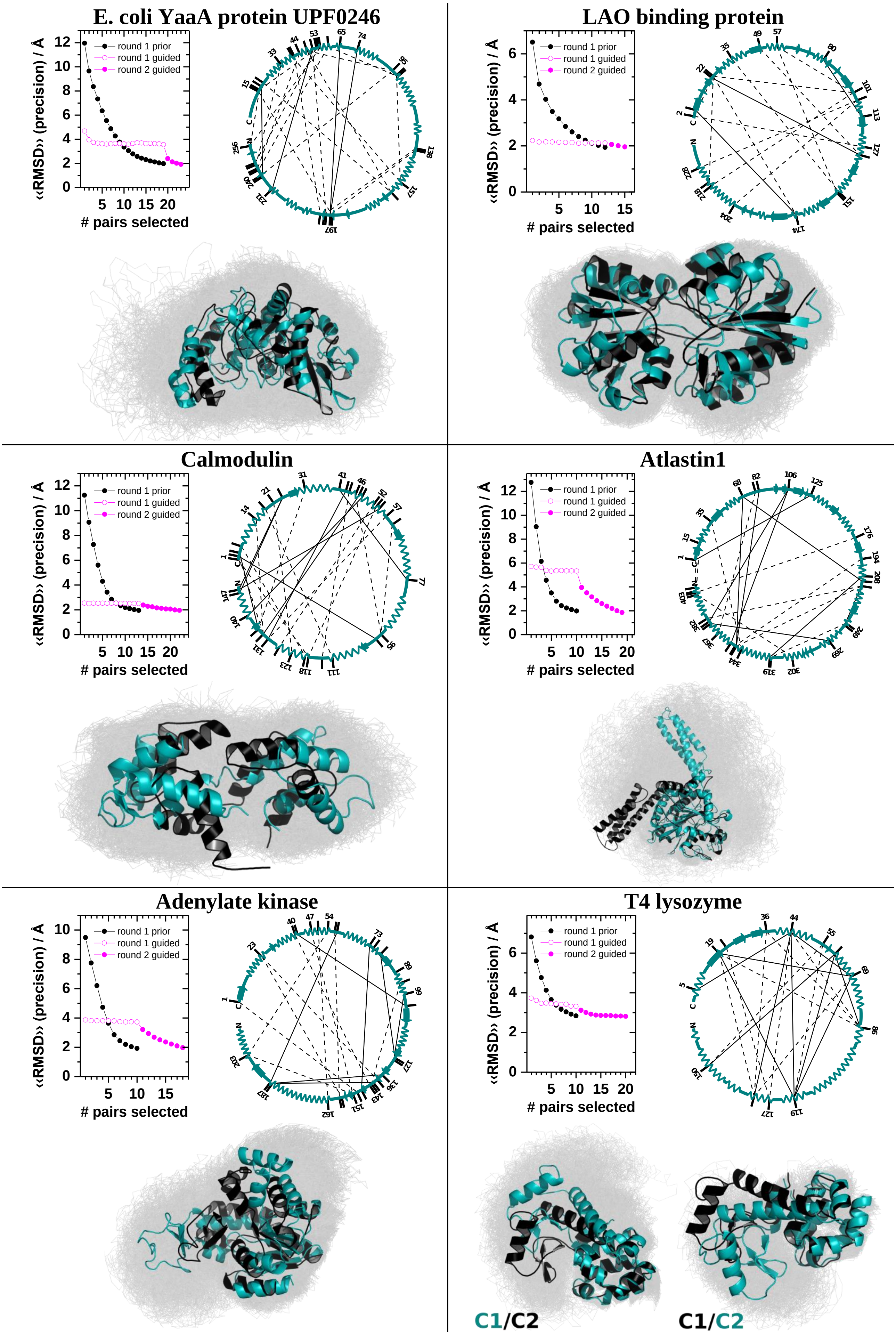

Supplementary Fig. 8 Expected precision and FRET pair networks of benchmarked proteins. Decay plots in the upper left corner of each block show expected precision depending on the number of FRET pairs measured: first round of selection based on initial (prior) ensemble is indicated by

black circles, second round of selection, based on the guided structural ensemble is indicated by full magenta circles, open magenta circles indicate, how pairs selected in the first round could discriminate the conformers obtained during guiding. One can see that pairs from the first round provide very little discrimination for the guided structures, as expected, since this information is already “used up”. FRET pair networks and secondary structures of corresponding seed conformers are shown to the upper right. Dashed lines indicate pairs selected in the first round, solid lines stand for the second round of selection. At the bottom initial conformational ensemble is shown in grey. Seed structure is shown in cyan and target conformer is in black. Source data are provided as a Source Data file.

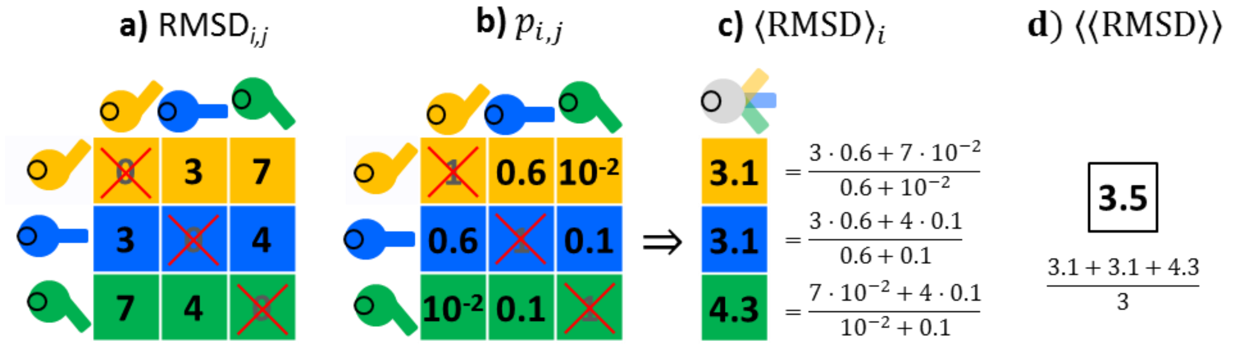

Supplementary Fig. 9 Calculation of expected precision.
For a given conformational ensemble of N conformers (here N = 3 for clarity: yellow, blue, green), the measure for expected precision ⟨⟨RMSD⟩⟩ is calculated: **(a)** The N x N matrix of pairwise RMSD values is computed, as are FRET observables for each conformer and **(b)** expected $p_{i,j}$ values (see **eq. 4**). Then, per-row weighted averages are taken to form **(c)** ⟨⟨RMSD⟩⟩$_i$, the elements of which are averaged to obtain **(d)** ⟨⟨RMSD⟩⟩.

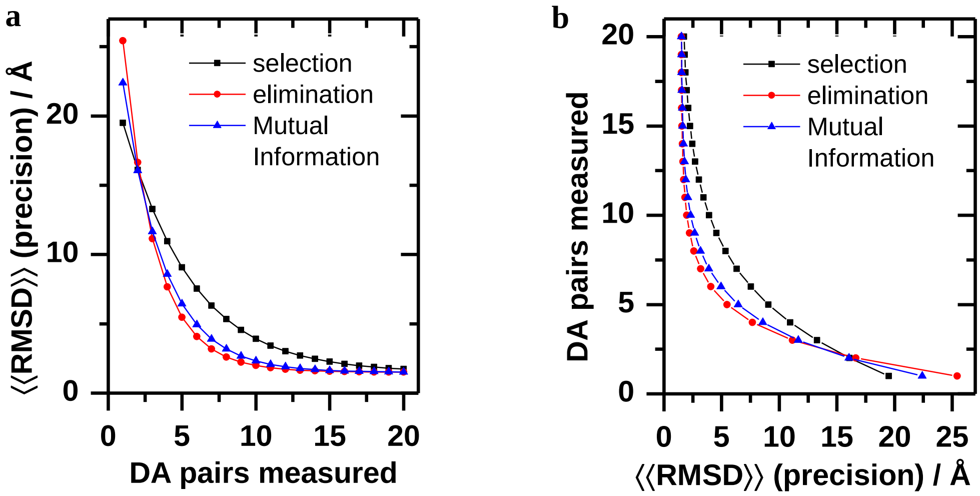

Supplementary Fig. 10 Optimal FRET pair selection algorithms.
(a) Dependence of expected precision on the measurement pair set size for different pair selection algorithms. The greedy pair **selection** algorithm (black, **Supplementary Note 4**) shows the lowest ⟨⟨RMSD⟩⟩ at a low number of measurements, although there the actual ⟨⟨RMSD⟩⟩ is high. The greedy pair **elimination** algorithm (red, **Supplementary Note 5**) yields the lowest ⟨⟨RMSD⟩⟩ except for a low number of measurements, however, this algorithm is also the most computationally demanding. The **mutual information**-based pair selection algorithm (blue, **Supplementary Note 6**) shows an intermediate behavior between the greedy pair selection and elimination algorithms; however, the greedy pair elimination algorithm is more computationally demanding by an order of magnitude. **(b)** Dependence of the measurement count on the desired precision ⟨⟨RMSD⟩⟩ (note, these are the inverse functions to those depicted in (**a**)). The steepness of the curves is system specific. The presented curves illustrate qualitative differences among selection algorithms. Source data are provided as a Source Data file.

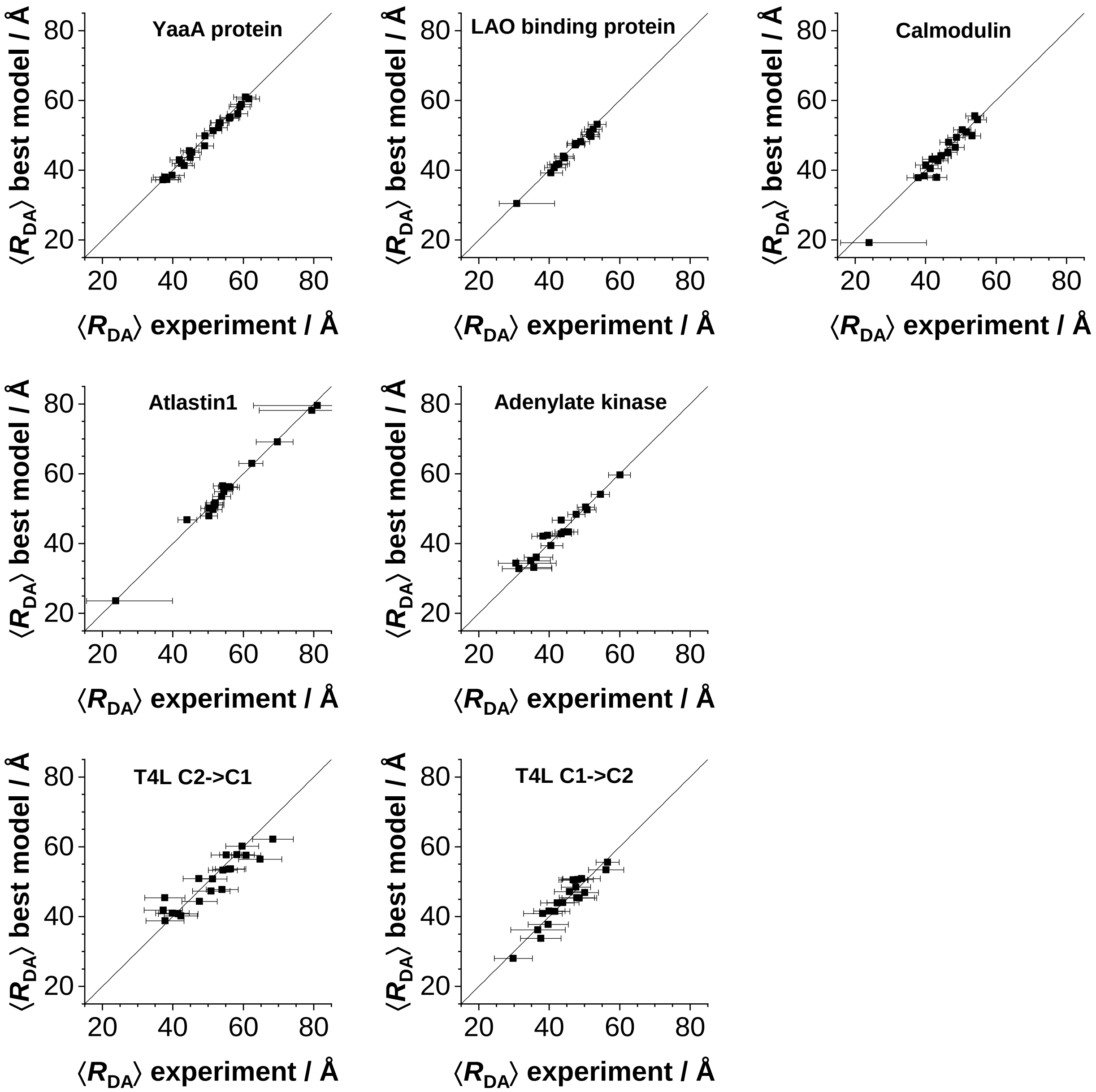

Supplementary Fig. 11 Measured FRET distances against predicted FRET distances for the best model (lowest χ_n^2). Error bars depict standard errors (see **Online Methods section 10**).

Supplementary Table 1 Selected FRET pairs and corresponding donor-acceptor averaged distances and errors for the target.

	**YaaA protein**		**LAO binding protein**		**Calmodulin**	
#	Pair	$\langle R_{DA}\rangle$ [+err, -err] / Å	Pair	$\langle R_{DA}\rangle$ [+err, -err] / Å	Pair	$\langle R_{DA}\rangle$ [+err, -err] / Å
1	138_201	58.4 [+2.7,-3]	49_131	40.4 [+3.3,-2.8]	31_135	48.4 [+2.4,-2.4]
2	139_198	56 [+2.5,-2.7]	57_152	41.4 [+3.1,-2.7]	53_118	54.7 [+2.5,-2.6]
3	15_197	43.2 [+2.8,-2.6]	35_151	44.3 [+2.7,-2.5]	18_111	23.9 [+16.2,-8.1]
4	16_256	45.4 [+2.6,-2.5]	105_228	47.6 [+2.5,-2.4]	21_123	43.5 [+2.8,-2.5]
5	16_54	42.5 [+2.9,-2.6]	105_204	53.6 [+2.4,-2.5]	46_95	39.5 [+3.5,-2.9]
6	18_239	41.9 [+3,-2.7]	23_101	52.5 [+2.4,-2.5]	52_127	
7	20_164	49 [+2.4,-2.4]	2_127	51.8 [+2.4,-2.5]	5_119	43.1 [+2.8,-2.6]
8	33_246	53.4 [+2.4,-2.5]	23_220	42.1 [+3,-2.7]	60_95	51.6 [+2.4,-2.4]
9	40_157	44.7 [+2.7,-2.5]	57_101	51.4 [+2.4,-2.4]	2_133	43.5 [+2.8,-2.5]
10	41_203	39.7 [+3.5,-2.9]	23_174	30.7 [+10.7,-4.9]	44_114	44.5 [+2.7,-2.5]
11	44_244	56.3 [+2.5,-2.8]	101_218	51.6 [+2.4,-2.4]	14_133	46.4 [+2.5,-2.4]
12	48_164	59.5 [+2.8,-3.1]	2_23	42.7 [+2.9,-2.6]	57_131	53.9 [+2.4,-2.6]
13	48_95	53.1 [+2.4,-2.5]	80_113	43.9 [+2.7,-2.5]	1_118	37.8 [+4,-3.2]
14	51_200	37.2 [+4.3,-3.3]	22_131	48.9 [+2.4,-2.4]	47_133	46.5 [+2.5,-2.4]
15	51_231	52.9 [+2.4,-2.5]	5_174	47.4 [+2.5,-2.4]	24_148	50.3 [+2.4,-2.4]
16	53_247	51.4 [+2.4,-2.4]			2_97	48.7 [+2.4,-2.4]
17	55_256	44.9 [+2.6,-2.5]			41_77	40 [+3.4,-2.9]
18	59_243	60.5 [+2.9,-3.3]			43_131	41.8 [+3,-2.7]
19	59_99	49.1 [+2.4,-2.4]			54_148	41.3 [+3.1,-2.7]
20	65_198	59.1 [+2.7,-3.1]			3_135	44.3 [+2.7,-2.5]
21	74_198	61.5 [+3,-3.5]			48_140	
22	95_240	38.3 [+3.9,-3.1]			24_147	53.1 [+2.4,-2.5]
23	98_157	37.6 [+4.1,-3.2]				

	**Atlastin1**		**Adenylate kinase**		**T4 lysozyme (C1→C2)**		**T4 lysozyme (C2→C1)**	
#	Pair	$\langle R_{DA}\rangle$ [+err, -err] Å	Pair	$\langle R_{DA}\rangle$ [+err, -err] Å	Pair	$\langle R_{DA}\rangle$ [+err, -err] Å	Pair	$\langle R_{DA}\rangle$ [+err, -err] Å
1	194_350	79.4 [+7.3,-14.9]	47_151	34.7 [+5.5,-3.8]	36_132	37.6 [+5.7,-5.7]	36_86	51.3 [+4,-4]
2	79_367	69.7 [+4.4,-6]	94_142	60.1 [+2.8,-3.2]	36_86	41.6 [+4.2,-4.2]	44_119	59.7 [+4.6,-4.6]
3	35_344	23.7 [+16,-8.2]	1_149	40.4 [+3.3,-2.8]	19_132	39.7 [+5.6,-5.6]	55_150	60.8 [+4.1,-4.1]
4	216_382	54.1 [+2.4,-2.6]	50_162	31.4 [+9.2,-4.7]	44_127	56.1 [+5,-5]	19_119	56.4 [+4.2,-4.2]
5	176_405	55.7 [+2.5,-2.7]	54_203	50.3 [+2.4,-2.4]	44_86	45.8 [+4.3,-4.3]	36_132	50.9 [+5.3,-5.3]
6	249_319	62.4 [+3.1,-3.7]	23_139	35.6 [+5,-3.6]	22_127	36.8 [+7.7,-7.7]	44_86	55.8 [+4.4,-4.4]
7	15_406	56.3 [+2.5,-2.8]	23_156		55_132	46.8 [+4,-4]	55_132	55.2 [+4.3,-4.3]
8	1_409	44 [+2.7,-2.5]	40_143	36.3 [+4.6,-3.4]	19_86	47.2 [+3.8,-3.8]	44_150	58.2 [+4.9,-4.9]
9	216_349	81 [+7.9,-18.1]	57_157	30.5 [+11.3,-5]	69_132	47.8 [+5,-5]	60_150	37.8 [+5.4,-5.4]
10	302_403		151_203	39.5 [+3.5,-2.9]	55_150	47.6 [+4.1,-4.1]	19_86	54.2 [+4,-4]
11	208_320	51.7 [+2.4,-2.4]	141_187	54.5 [+2.5,-2.6]	60_150	48.5 [+4.9,-4.9]	60_86	54 [+4.5,-4.5]
12	269_377	52 [+2.4,-2.5]	79_127	43.4 [+2.8,-2.6]	8_86	38.2 [+5.5,-5.5]	55_119	68.4 [+5.8,-5.8]
13	106_354	54.4 [+2.5,-2.6]	73_147	45.4 [+2.6,-2.4]	44_119	50.1 [+3.8,-3.8]	44_132	64.8 [+6.1,-6.1]
14	82_349	50.3 [+2.4,-2.4]	75_89	44.1 [+2.7,-2.5]	60_86	43.9 [+4.5,-4.5]	69_119	39.9 [+4.7,-4.7]
15	68_212	50.2 [+2.4,-2.4]	41_104	47.6 [+2.5,-2.4]	44_69	29.8 [+5.4,-5.4]	60_119	47.4 [+4.4,-4.4]
16	106_379		136_187	50.8 [+2.4,-2.4]	60_132	49.2 [+5.3,-5.3]	8_86	47.6 [+5,-5]
17	1_125	53.8 [+2.4,-2.6]	58_188	38.2 [+3.9,-3.1]	5_44	42.3 [+4.7,-4.7]	69_132	37.3 [+5.4,-5.4]
18	216_251	52 [+2.4,-2.5]	99_128	43.4 [+2.8,-2.6]	69_119	40 [+4.4,-4.4]	5_44	42.3 [+4.7,-4.7]
19	68_349	51.5 [+2.4,-2.4]			44_150	48.1 [+4.4,-4.4]	60_132	37.7 [+5.7,-5.7]
20					55_119	56.6 [+3.2,-3.2]	22_127	41.5 [+5.6,-5.6]

Lists of selected FRET pairs for each of the benchmarked proteins. Donor and acceptor residue IDs are indicated for each pair. $\langle R_{DA}\rangle$ stands for the average donor-acceptor distance. Pairs are ordered by relevance, starting from the most relevant. Pairs selected additionally for cross-validation are underlined. Reference distances and corresponding errors are provided unless the labeling site is inaccessible in the reference conformer; in the latter case, this distance pair was not included in the further analysis. In the case of T4 lysozyme experimentally measured values are reported, for other proteins simulated data is provided. For the generation of in silico FRET data, error of FRET efficiency of 0.06 was assumed and propagated to the inter-dye distance errors. This magnitude of error is typical for FRET measurements according to the multi-laboratory benchmark study[2].

Supplementary Table 2 List of primers used within this work.

T4Lfor and T4Lrev were used for subcloning into the pet11a vector. Note that T4Lfor lies within the backbone of pet11a to have sufficient distance to the first mutation site (amino acid residue 5).

Primer*	Sequence (5'->3')
**T4Lfor**	GGAATGGTGCATGCAAGGAGATGG
**T4Lend****	GCC*GGATCC*TTATAGATTTTATACGC
**E5Amber for**	ATGAATATATTTTAGATGTTACGTATAGAT
**E5Amber rev**	ATCTATACGTAACTACTAAAATATATTCAT
**R8Amber for**	AATATATTTGAAATGTTATAGATAGATGAACGTCTTAGA
**R8Amber rev**	TCTAAGACGTTCATCTATCTATAACATTTCAAATATATT
**K19Amber for**	CTTAGACTTAAAATCTATTAGGACACAGAAGGCTATTAC
**K19Amber rev**	GTAATAGCCTTCTGTGTCCTAATAGATTTTAAGTCTAAG
**E22Amber for**	AAAATCTATAAAGACACATAGGGCTATTACACTATTGGC
**E22Amber rev**	GCCAATAGTGTAATAGCCCTA GTGTCTTTATAGATTTT
**S36Amber for**	GGTCATTTGCTTACAAAATAGCCATCACTTAATGCTGCT
**S36Amber rev**	AGCAGCATTAAGTGATGGCTATTTTGTAAGCAAATGACC
**S44Amber for**	TCACTTAATGCTGCTAAATAGGAATTAGATAAAGCTATT
**S44Amber rev**	AATAGCTTTATCTAATTCCTATTTAGCAGCATTAAGTGA
**S44C for**	TCACTTAATGCTGCTAAATGTGAATTAGATAAAGCTATT
**S44C rev**	AATAGCTTTATCTAATTCACATTTAGCAGCATTAAGTGA
**N55Amber for**	GCTATTGGGCGTAATACTTAGGGTGTAATTACAAAAGAT
**N55Amber rev**	ATCTTTTGTAATTACACCCTAAGTATTACGCCCAATAGC
**K60Amber for**	ACTAATGGTGTAATTACATAGGATGAGGCTGAAAAACTC
**K60Amber rev**	GAGTTTTTCAGCCTCATCCTATGTAATTACACCATTAGT
**Q69Amber for**	GCTGAAAAACTCTTTAATTAGGATGTTGATGCTGCTGTT
**Q69Amber rev**	AACAGCAGCATCAACATCCTAATTAAAGAGTTTTTCAGC
**Q69C for**	GCTGAAAAACTCTTTAATTGTGATGTTGATGCTGCTGTT
**Q69C rev**	AACAGCAGCATCAACATCACAATTAAAGAGTTTTTCAGC
**D70Amber for**	GAAAAACTCTTTAATCAGTAGGTTGATGCTGCTGTTCGC
**D70Amber rev**	GCGAACAGCAGCATCAACCTACTGATTAAAGAGTTTTTC
**P86C for**	AGAAATGCTAAATTAAAATGTGTTTATGATTCTCTTGAT
**P86C rev**	ATCAAGAGAATCATAAACACATTTTAATTTAGCATTTCT
**R119C for**	GGATTTACTAACTCTTTATGTATGCTTCAACAAAAACGC
**R119C rev**	GCGTTTTTGTTGAAGCATACATAAAGAGTTAGTAAATCC
**D127C for**	CTTCAACAAAAACGCTGGTGTGAAGCAGCAGTTAACTTA
**D127C rev**	TAAGTTAACTGCTGCTTCACACCAGCGTTTTTGTTGAAG
**N132C for**	TGGGATGAAGCAGCAGTTTGTTTAGCTAAAAGTAGATGG
**N132C rev**	CCATCTACTTTTAGCTAAACAAACTGCTGCTTCATCCCA
**R137E for**	CAATTGAATCGATTTTCA CTTACCATATTAGTTTGTGGA
**R137E rev**	GTTAACTTAGCTAAAAGTGAATGGTATAATCAAACACCT
**I150C for**	AATCGCGCAAAACGAGTCTGTACAACGTTTAGAACTGGC
**I150C rev**	GCCAGTTCTAAACGTTGTACAGACTCGTTTTGCGCGATT

*The underlined nucleotides mark the mutation side.

**The italic nucleotides mark the restriction enzyme recognition site

Supplementary Table 3 Site-specific residual anisotropies for donor and acceptor dyes of T4 lysozyme.

Donor, Alexa488		Acceptor, Alexa647	
**Residue sequence number**	**r_∞/r_0**	**Residue sequence number**	**r_∞/r_0**
5	0.72	44	0.43
8	0.67	86	0.49
19	0.43	119	0.55
22	0.58	69	0.57
36	0.55	150	0.61
44	0.51	127	0.68
55	0.33	132	0.69
60	0.54		
69	0.44		
70	0.46		

Ratio of the residual anisotropy, r_∞, determined experimentally by analysis of time- and polarization resolved fluorescence decays of fluorescent labeled T4 lysozyme over fundamental anisotropy r_0 = 0.38 of the dyes.

Supplementary Note 1: System selection and geometric modeling justification.

For this benchmark study, we selected systems where similar approaches have been applied[3-5]. These systems are representative molecules of different sizes (148 to 409 aa), they reflect different interconversion motions (hinge bending, shear, twist), and the mode of interaction with target molecules is different (Induced fit or conformational selection).

Because NMSim samples geometrically allowed (considering covalent and non-covalent bond constraints) conformations of proteins, there is less emphasis on the mode of motion or interactions. Hence, even low populated states with high energy and non-physiological states as in the case of induced fit are allowed, because the sampling over these geometric models generates flat energy landscapes, reaching to states that traditional MD simulations would not allow. The drawback is that the relative energy between states is lost. Therefore, with NMSim, it is possible to reach induced fit configurations even in the absence of ligands; highlighting the predictive nature of NMsim over traditional MD simulations, which require more complex simulations and are more computational expensive. For example, the ligand bound form of Calmodulin is reached even when the seed structure corresponds to the Apo-state.

Supplementary Note 2: Dye models in the simulations

Accessible volume (AV) simulations were successfully used to estimate the average donor-acceptor distances $\langle R_{DA} \rangle$ from structural models of RNA and DNA[6]. An AV is the sterically allowed space of the dye molecule attached to the protein as calculated by the FPS program[7]. In proteins dyes can be trapped on the protein surface to a significant extent (see **Supplementary Table 3**). To account for this, we used the Accessible and Contact Volume (ACV) dye model for all simulations[8]. The surface areas of the ACVs were considered separately using the anisotropy values determined from experiment. For that, we defined contact volume as the part of the AV which is closer than R_{CV} = 3 Å from the protein surface. Population fraction of the dye within the contact volume is assigned to a higher value equal to the experimental ratio of residual anisotropy over fundamental anisotropy r_∞/r_0 of the corresponding labelling position as determined from the T4L experiments[8] (see **Supplementary Table 3**).

T4L was labeled by Alexa488 with a C5-hydroxylamine linker (Donor), which is coupled to the unnatural amino acid p-acetylphenylalanine, and Alexa647 with a C2-maleimide linker (Acceptor), which is coupled to cysteine (see **Methods section 9**). Despite the different coupling chemistry and distinct fluorophores a single set of dye parameters is most suitable to describe the experiments. In the simulations these dye/linker pairs were approximated as flexible tubes with width of L_{width} = 2.5 Å and length of L_{link} = 21.0 Å. The fluorophore moieties were approximated by spheres with a radius of R_{dye} = 3.5 Å. The same dye parameters were also used for the simulation of FRET data.

In the simulated data constant value of r_∞/r_0 = 0.3 was used to mimic a typical fraction of trapped dye. In the simulated data, the uncertainty level of average FRET efficiency standard error was constant ($E = E_{\mathrm{ref}} \pm 0.06$), which corresponds to typical magnitude of the error in such experiments. This leads to asymmetric uncertainties of the average donor-acceptor distances ΔR_{ref}. Depending on the target FRET efficiency E_{ref}, uncertainties ΔR_{ref} vary in the range from 2.0 to 20 Å (see **Supplementary Table 1**).

**Parameter**	**Value**
L_{link}	21.0 Å
L_{width}	2.5 Å
R_{dye}	3.5 Å
R_{CV}	3.0 Å
Grid resolution	0.9
Förster radius	52.0 Å
Allowed sphere radius	1.5 Å
Used for simulated data:	
Efficiency Error	0.06
r_∞/r_0	0.3

Supplementary Note 3: **Local Distance Difference Test (lDDT)**

In order to compare structural similarity and accuracy of structural models, we use the local Distance Difference Test (lDDT) superposition-free score[9], which is has been applied as one of the structural similarity scores in Critical Assessment of techniques for protein Structure Prediction (CASP) competitions[10]. Compared to the root-mean-square deviation (RMSD) criterion, lDDT puts extra emphasis on local model quality like secondary structure and does not require superposition of the tested and reference conformers.

Standard lDDT computes distances between atoms in a model, but no further than 15 Å apart and only if atoms do not belong to the same residue; the same set of distances is calculated for the reference model. Both sets are compared to determine, how many distances are preserved. The distance is considered preserved if it is within a certain tolerance threshold from the corresponding reference distance. Standard lDDT calculates the average over the individual fractions of preserved distances for threshold values of 0.5 Å, 1 Å, 2 Å, and 4 Å. In this study, we focus on the backbone conformation and only use C_α atoms to calculate the lDDT score.

Supplementary Note 4: Pseudocode for greedy FRET pair selection algorithm.

```
def greedySelection(RMSD_target=2.0):
    residues = range(1,len(protein))
    pairs = combinations(residues, 2) #all donor-acceptor pairs
    selected = []
    RMSDmin = float("inf")
    while RMSDmin > RMSD_target:
        RMSDmin = float("inf")
        bestPair = pairs[0]
        for pair in pairs:
            RMSD = rmsd_ave(ensemble,selected+[pair])
            if RMSD<RMSDmin:
                bestPair = pair
                RMSDmin = RMSD
        selected.append(bestPair)
        print(len(selected), bestPair, RMSDmin)
```

Let us assume that the protein of interest has 100 amino acids. In this case, the number of possible donor-acceptor combinations is:

$$N_{\text{pairs}} = C_{n,k} = \binom{n}{k} = \frac{n!}{k!\,(n-k)!} = \frac{100!}{2!\,(100-98)!} = 4950$$

Many of these pairs cannot be labeled, however, and in practice will be excluded from selection. To select the first informative FRET pair, the algorithm iterates through all of the possible N_{pairs} donor-acceptor pairs. For each potential donor-acceptor pair, the value of expected precision $\langle\langle \text{RMSD} \rangle\rangle_{[\text{pair}]}$ is calculated (eq. 6). This value quantifies, how precisely one can determine a conformation out of an ensemble, using the specified pair or set of pairs. Then $\langle\langle \text{RMSD} \rangle\rangle_{[\text{pair}]}$ values, calculated for each pair independently, are ranked and the FRET pair, that corresponds to the lowest $\langle\langle \text{RMSD} \rangle\rangle_{[\text{pair1}]}$ is saved as the first most informative FRET pair. Let's say, for example, that the first most informative pair is between donor at position 10 and acceptor at position 90: [D10_A90].

To select a second pair, the procedure is repeated from the beginning with the exception, that now $\langle\langle \text{RMSD} \rangle\rangle_{[\text{D10\_A90;pair2}]}$ values are calculated for the *sets* of pairs, composed of the first informative FRET pair plus the iterated pair. The best second pair corresponds to the lowest $\langle\langle \text{RMSD} \rangle\rangle_{[\text{D10\_A90;pair2}]}$. The procedure is repeated to select additional pairs, until the desired expected precision $\langle\langle \text{RMSD} \rangle\rangle_{[\text{pair1}, \dots, \text{pairN}]}$ is obtained.

Supplementary Note 5: Pseudocode for greedy FRET pair elimination algorithm.

```
def greedyElimination(RMSD_target=2.0):
    residues = range(1, len(protein))
    selected = combinations(residues, 2)
    RMSDmin = float("inf")
    while RMSDmin > RMSD_target:
        RMSDmin = float("inf")
        for pair in selected:
            pairs = copy(selected)
            pairs.remove(pair)
            RMSD = rmsd_ave(ensemble, pairs)
            if RMSD<RMSDmin:
                worstPair = pair
                RMSDmin = RMSD
        selected.remove(worstPair)
        print(len(selected), worstPair, RMSDmin)
```

The greedy elimination algorithm works very similarly to the greedy elimination, except we start with the set of N_{pairs} FRET pairs and remove them one by one starting from the least informative. Pair is defined as less informative, if without it the ⟨⟨RMSD⟩⟩ increases as little as possible.

Supplementary Note 6: Pseudocode for mutual information-based FRET pair selection algorithm.

```
def MI_selection(RMSD_target=2.0):
    residues = range(1,len(protein))
    pairs = combinations(residues, 2)
    #Shannon entropies for each pair
    entropies = [entropy(pair) for pair in pairs]
    iBest = argmax(entropies)
    selected = [pairs[iBest]]

    RMSD = rmsd_ave(ensemble, selected)
    print(1, selected[0], RMSD)
    while RMSD > RMSD_target:
        minCHlist = [] #conditional entropies
        for pair in pairs:
            condHlist = []
            for prev in selected:
                condHlist.append(conditionalEntropy(pair,prev))
            minCHlist.append(min(condHlist))
        iMaxCH = minCHlist.index(max(minCHlist))
        bestPair = pairs[iMaxCH]
        selected.append(bestPair)
        RMSD = rmsd_ave(ensemble, selected)
        print(len(selected), bestPair, RMSD)
```

In the mutual information-based pair selection, first we select the FRET pair with the highest Shannon entropy.

To select the second pair, conditional entropy of each pair is calculated against the first pair. The pair, that has the highest conditional entropy with respect to the first pair is considered the most informative. This means, that this pair adds the most additional information.

To select the third pair, we calculate two conditional entropies for each potential pair: one against the first selected pair and one against the second selected pair. Minimal value of this two is used as the conditional entropy associated to the given potential pair. The pair with the highest conditional entropy is selected as the third most informative. The third pair adds the most information in addition to the first and the second. Here we approximate the multivariate conditional entropy of the triplet by a minimum of pairwise conditional entropies.

We reiterate this procedure until the desired expected precision $\langle\langle \text{RMSD} \rangle\rangle$ is achieved.

Supplementary Note 7: **NMSim coarse-grained simulations**

Unbiased and FRET-guided structural ensembles were generated by the NMSim software[1] (http://www.nmsim.de). For unbiased NMSim simulations ten simulations generating 10,000 conformations (steps) each were performed, starting from the seed structure and using default parameters for sampling of large-scale motions. These trajectories are clustered and serve as initial candidates.

In the case of FRET-guided simulations, the same NMSim parameters were used. Additionally, a Monte Carlo Metropolis-Hastings annealing procedure was applied, in which FRET χ_r^2 of the conformation is used as the guiding potential. A single FRET-guided NMSim simulation of 10,000 steps contains two annealing cycles, such that effective temperature varies from $kT = 0$ to $kT = kT_{\text{max}}$ and back to $kT = 0$. For each seed structure, five FRET-guided NMSim simulations were performed for $kT_{\text{max}} = 0.1$ units of χ_r^2 and another five for $kT_{\text{max}} = 1.0$ units of χ_r^2.

**Parameter**	**Value**
E-cutoff for H-bonds	-1.0
Hydrophobic cutoff	0.35
Hydrophobic method	3
No. of sim. cycles	10000
No. of NMSim cycles	1
NM mode range	1-5
Step size	0.5
C-alpha Cutoff	10
kT_{max}	1.0; 0.1

References

1. Ahmed, A., Rippmann, F., Barnickel, G. & Gohlke, H. A normal mode-based geometric simulation approach for exploring biologically relevant conformational transitions in proteins. *J. Chem. Inf. Model.* **51**, 1604-1622 (2011).
2. Hellenkamp, B. *et al.* Precision and accuracy of single-molecule FRET measurements-a multi-laboratory benchmark study. *Nat. Meth.* **15**, 669-676 (2018).
3. Cavasotto, C. N., Kovacs, J. A. & Abagyan, R. A. Representing receptor flexibility in ligand docking through relevant normal modes. *J. Am. Chem. Soc.* **127**, 9632-9640 (2005).
4. Ahmed, A. & Gohlke, H. Multiscale modeling of macromolecular conformational changes combining concepts from rigidity and elastic network theory. *Proteins* **63**, 1038-1051 (2006).
5. Tama, F. & Sanejouand, Y. H. Conformational change of proteins arising from normal mode calculations. *Protein Eng.* **14**, 1-6 (2001).
6. Sindbert, S. *et al.* Accurate distance determination of nucleic acids via förster resonance energy transfer: implications of dye linker length and rigidity. *J. Am. Chem. Soc.* **133**, 2463-2480 (2011).
7. Kalinin, S. *et al.* A toolkit and benchmark study for FRET-restrained high-precision structural modeling. *Nat. Meth.* **9**, 1218-1227 (2012).
8. Dimura, M. *et al.* Quantitative FRET studies and integrative modeling unravel the structure and dynamics of biomolecular systems. *Curr. Opin. Struct. Biol.* **40**, 163-185 (2016).
9. Mariani, V., Biasini, M., Barbato, A. & Schwede, T. lDDT: a local superposition-free score for comparing protein structures and models using distance difference tests. *Bioinformatics* **29**, 2722-2728 (2013).
10. Mariani, V., Kiefer, F., Schmidt, T., Haas, J. & Schwede, T. Assessment of template based protein structure predictions in CASP9. *Proteins* **79**, 37-58 (2011).
